# The accuracy of Acuros XB algorithm for radiation beams traversing a metallic hip implant — comparison with measurements and Monte Carlo calculations

**DOI:** 10.1120/jacmp.v15i5.4912

**Published:** 2014-09-08

**Authors:** Jarkko Ojala, Mika Kapanen, Petri Sipilä, Simo Hyödynmaa, Maunu Pitkänen

**Affiliations:** ^1^ Department of Oncology Unit of Radiotherapy, Tampere University Hospital Tampere Finland; ^2^ Department of Medical Physics Medical Imaging Center, Tampere University Hospital Tampere Finland; ^3^ Radiation Practices Regulation Radiotherapy and Nuclear Medicine, STUK– Radiation and Nuclear Safety Authority Helsinki Finland

**Keywords:** Monte Carlo dose calculation, Acuros, AXB, implant, photon beam radiotherapy

## Abstract

In this study, the clinical benefit of the improved accuracy of the Acuros XB (AXB) algorithm, implemented in a commercial radiotherapy treatment planning system (TPS), Varian Eclipse, was demonstrated with beams traversing a high‐Z material. This is also the first study assessing the accuracy of the AXB algorithm applying volumetric modulated arc therapy (VMAT) technique compared to full Monte Carlo (MC) simulations. In the first phase the AXB algorithm was benchmarked against point dosimetry, film dosimetry, and full MC calculation in a water‐filled anthropometric phantom with a unilateral hip implant. Also the validity of the full MC calculation used as reference method was demonstrated. The dose calculations were performed both in original computed tomography (CT) dataset, which included artifacts, and in corrected CT dataset, where constant Hounsfield unit (HU) value assignment for all the materials was made. In the second phase, a clinical treatment plan was prepared for a prostate cancer patient with a unilateral hip implant. The plan applied a hybrid VMAT technique that included partial arcs that avoided passing through the implant and static beams traversing the implant. Ultimately, the AXB‐calculated dose distribution was compared to the recalculation by the full MC simulation to assess the accuracy of the AXB algorithm in clinical setting. A recalculation with the anisotropic analytical algorithm (AAA) was also performed to quantify the benefit of the improved dose calculation accuracy of type ‘c’ algorithm (AXB) over type ‘b’ algorithm (AAA). The agreement between the AXB algorithm and the full MC model was very good inside and in the vicinity of the implant and elsewhere, which verifies the accuracy of the AXB algorithm for patient plans with beams traversing through high‐Z material, whereas the AAA produced larger discrepancies.

PACS numbers: 87.55.‐x, 87.55.D‐, 87.55.K‐, 87.55.kd, 87.55.Qr

## I. INTRODUCTION

Patients undergoing radiotherapy of the pelvic region make up a significant portion of all the patients treated with radiotherapy. During past several decades, the number of people with implanted hip prostheses has increased globally. Along with this increase, the average life expectancy has also continuously increased which, in combination with improved diagnostic methods, have led to increased cancer detection rates. All these factors together have resulted in increased number of radiotherapy patients having prosthetic devices implanted in their bodies.^1^, ^2^ There are three different types of hip implant materials. The most common alloy contains cobalt, chromium, and molybdenum, but there are also implants made of titanium‐aluminum‐vanadium alloy and stainless steel. Common for all these high‐density and atomic number (Z) material combinations is the corrosion and fatigue resistance and mechanical strength. A comprehensive report on dosimetric considerations for patients with hip prostheses undergoing pelvic irradiation was released by the AAPM task group in 2003.^(3)^

For the radiotherapy treatment planning of patients with unilateral or bilateral implants, two major challenges have been recognized. Firstly, the hip implant made of high‐Z material (in this work materials with greater Z than found in human tissues are considered high‐Z materials) generates considerable artifacts in the computed tomography (CT) images that have an essential role in radiotherapy dose calculation. To reduce the effect of artifacts (i.e., incorrect Hounsfield unit (HU) values), a multitude of various metal artifact reduction/removal (MAR) techniques have been developed or a constant HU value is assigned, which represents an average HU value of the tissue in the region in question. Also the extended HU value scale has been developed to allow the differentiation of high‐Z materials from tissues, and even identify various metals and alloys used in implants.^4^, ^5^

Secondly, the presence of implanted high‐Z material generates a notable attenuation to the beam in the shadow of the implant, which affects the dose distribution in that region, and also a dose peak upstream from the material surface caused by backscatter. The production of clinically acceptable dose distributions in patient volumes with high‐Z materials has been almost an impossible task for the commercial dose calculation algorithms to date.^(2)^ Type ‘a’ algorithms,^(6)^ based for example on pencil beam principles, underestimate the dose attenuation in the hip implant. When applying a typical four‐field box technique, used in the past with 6 MV photon beams to prostate cancer patients with unilateral or bilateral hip implants, the overestimation of the target dose has been 10%^(7)^ and 14%,^(1)^ respectively. Type ‘b’ algorithms (such as superposition/convolution techniques^(6)^) that account for the secondary electron transport to varying degrees have produced better congruence with reference methods in the presence of high‐Z materials than type ‘a’ algorithms, as demonstrated by Glide‐Hurst et al.,^(5)^ Keall et al.,^(8)^ and Wieslander and Knöös,^(9)^ for example. As type ‘c’ algorithms we define those most recent generation commercial algorithms that apply fast Monte Carlo codes or grid‐based linear Boltzmann transport equation (LBTE) solver that are able take into account high‐Z materials in dose calculation, and with which the user may choose the dose report mode between dose‐to‐medium and dose‐to‐water. With type ‘c’ algorithms, the dose calculation accuracy with high‐Z materials has been improved near to the level of full MC methods and/or measurements that have been used as reference dose distributions, which has been shown for grid‐based LBTE solver by Failla et al.^(10)^ and Lloyd and Ansbacher.^(11)^ The Acuros XB (AXB) algorithm by Varian Medical Systems, Inc. (VMS) (Palo Alto, CA) is a grid‐based LBTE solver that is a part of the Eclipse treatment planning system (TPS) (VMS). It was the first commercial algorithm that was reported to be able to model the sharp dose gradients in the vicinity of low‐Z/high‐Z material interface.^10^, ^11^ Common for many studies is the conclusion that the full MC calculations are able to simulate dose distributions featuring high‐Z materials accurately, which is consistent with the deduction that the MC methods can be used as a primary reference for various purposes, but only if cautiously commissioned.^3^, ^12^

In the context of this work there are no studies where type ‘c’ commercial dose calculation algorithms have been benchmarked with measurements in CT‐based phantoms and in combination with modern treatment techniques, such as volumetric‐modulated arc therapy (VMAT), with beams traversing the implant. In the study by Lloyd and Ansbacher,^(11)^ the measurements were performed in a slab phantom and the MC simulations in the corresponding non‐CT‐based virtual phantom. The results obtained in the phantom geometry cannot be straightforwardly applied in clinical setting, and also the effect of artifacts in CT image dataset should be evaluated. Regardless of this, the study revealed that the type ‘b’ algorithm by VMS (the anisotropic analytical algorithm (AAA)) underestimated the water/high‐Z material interface peak dose by 20% for a single 18 MV photon beam; the underestimation for a single 6 MV photon beam being as large as 17% at the interface upstream and lateral from the high‐Z insert in the phantom. Downstream the high‐Z insert similar levels of dose overestimation by the AAA were found. In patient cases, the full potential of the type ‘c’ algorithm in the presence of hip implant has not been revealed, since only post‐target parts of the static beams have traversed the implant in the existing studies. On the other hand, in the study by Failla et al.,^(10)^ only computational methods were used to verify the accuracy of the AXB algorithm. Due to the previously mentioned partially unsolved challenges, for patients with hip implants it has been common to avoid beam directions traversing the implant.^(3)^ This is the first study assessing the accuracy of the AXB algorithm applying VMAT technique compared to full MC simulations that are validated against measurements in phantom geometry.

To address these issues, in the first phase of this study the AXB algorithm is benchmarked against point dosimetry, film dosimetry, and full MC method in an anthropometric phantom filled with water and with a unilateral hip implant. To quantify the effect of the CT artifacts, the most prevalent being dark and white streak artifacts outside high‐Z material and incorrect HU values inside the high‐Z material, calculations are performed with the original CT dataset and corrected CT dataset, where constant HU values are assigned to neglect the effect of artifacts. To demonstrate the clinical benefit of the AXB algorithm, in the second phase of this study a clinical treatment plan is prepared for a prostate cancer patient with a unilateral hip implant using VMAT technique with avoidance sectors and static beams passing through the implant (referred to as hybrid technique). The dose distribution is compared to the full MC simulation to assess the accuracy of the AXB algorithm in the clinical setting with beams traversing the implant. In addition, the AAA is included in patient plan comparison to quantify the differences between type ‘b’ algorithm (AAA) and full MC simulation, and to quantify the benefit of the improved dose calculation accuracy of type ‘c’ algorithm (AXB) over type ‘b’ algorithm (AAA).

## II. MATERIALS AND METHODS

### A. The TPS dose calculation algorithms

In this study, version 10.0 of the Eclipse TPS and version 10.0.28 of the AXB algorithm and the AAA were used. The linear accelerator (linac) used in this study was the Varian Clinac iX (2300 C/D) linac equipped with Millennium 120 MLC (5 mm thick leaves at isocenter plane around beam central axis (CAX)). The configuration of the algorithm was performed strictly following manufacturer's manuals and its recommendations and selecting default configuration settings. The effective target spot sizes were 1.0 and 0.0 mm for the AXB algorithm and the AAA, respectively. The MLC leaf transmission was 1.4% and dosimetric leaf gap was 0.19 cm. The measurements were performed with IBA unshielded stereotactic semiconductor field detector (model DEB050; IBA Dosimetry AB, Sweden) for 2 × 2 to $4\;\times \;4\;{\text{cm}}^{2}$ fields and with PTW TM31002 Semiflex 0.125 cm^3^ ionization chamber (IC) (PTW Freiburg GmbH, Germany) for 3 × 3 to $40\;\times \;40\;{\text{cm}}^{2}$ fields using a motorized scanning system in an MP3 water phantom (PTW Freiburg GmbH). The smallest field size in output factor configuration was $2\;\times \;2\;{\text{cm}}^{2}$ and the measurement results were “daisy chained”, as presented by Dieterich and Sherouse.^(13)^ The AXB algorithm is a non‐analytical model‐based algorithm and represents the most recent generation of clinical dose calculation algorithms. It solves deterministically the coupled system of LBTEs. The radiation beam propagation in the treatment head is modeled with the multiple source model implemented originally for the AAA.^(14)^ The source model contains subsources for primary photons, extrafocal photons, and electron contamination. The model is fine‐tuned using the user‐supplied beam measurement data during the beam configuration.

In the radiation transport and dose calculation in the patient the following steps are performed: 1) transport of source model fluence onto the patient; 2) calculation of scattered photon fluence in the patient; 3) calculation of scattered electron fluence in the patient; and 4) dose calculation. In the heterogeneity correction, the AXB algorithm explicitly models the physical interactions of radiation with matter and, thus, the report mode for the final dose distribution is referred to as dose‐to‐medium in medium $\left(D_{m,m}\right)$. Although the AXB algorithm inherently calculates $D_{m,m}$, the dose distributions can be converted to dose‐to‐water in medium $\left(D_{w,m}\right)$, which is done by replacing the medium‐based fluence‐to‐dose response function used in absorbed dose calculation with a water‐based response function.^(10)^

The AAA is a semianalytical model‐based algorithm, although its core is built on exploiting pencil beams. The pencil beams are determined from Monte Carlo simulations fitted to user‐supplied beam measurements, after which the multiple source model is determined. Heterogeneity correction in the AAA takes into account (to some extent) the scattered radiation from the surroundings of the calculation point (i.e., in the lateral scaling of the medium, it applies six independent exponential functions to account for the lateral transport of energy with varying densities).^15^, ^16^, ^17^ In the AAA, the dose report mode is $D_{w,m}$, but the dose results are based on electron density‐based corrections applied to dose kernels calculated in water.^10^, ^18^, ^19^

### B. The MC model

“Full” MC simulations were performed with the BEAMnrc code package (V4‐2.4.0, or BEAMnrc 2013), which uses the EGSnrc MC code that simulates coupled electron–photon transport. The EGSnrc‐based phantom dose calculation is performed with DOSXYZnrc, also included in the BEAMnrc code package.^(20)^ The geometry model of the linac treatment head was based on the same Varian Clinac iX as in Materials and Methods section A. The MC model was based on the earlier work by the authors.^21^, ^22^

The nominal photon beam energy was 6 MV. The simulation of beam generation and beam transport in the treatment head was divided into two phases to allow the absolute dose calibration of the MC model, following the technique by Popescu et al.^(23)^ The iterative initial electron beam tuning process and beam parameter selection are discussed in Ojala et al.^21^, ^22^ The first phase simulation through the static components of the treatment head had to be performed only once, with the number of particle histories of $10\;\times \;10^{9}$. The resulting particle data were collected into a phase space file, which was used as source for the second phase simulation through beam‐modifying components. This part was simulated as BEAMnrc shared library that was dynamically loaded by DOSXYZnrc code at run time. DOSXYZnrc source 20 in combination with synchronized beam‐modifying components as shared library allow the simulation of plans containing multiple fields or field segments (such as VMAT) in a single run.^(24)^ The plan parameters (e.g., jaw and MLC apertures, gantry, collimator settings for each control point) were exported from the TPS and converted to the form required by the MC code package. The electron and photon transport cutoff parameters used in the second phase and DOSXYZnrc simulations were ECUT = AE = 0.521 MeV and PCUT = AP = 0.01 MeV for the phantom study, but ECUT = AE = 0.700 MeV for the patient plan. The value for ECUT for MC represents the cutoff of total (rest + kinetic) energy of the electron and it was chosen to provide accurate secondary electron transport. The cutoff value for electron kinetic energy for the AXB algorithm was 0.500 MeV (1.011 MeV total energy), which is unmodifiable by the user. Other EGSnrc parameters were the same as in the earlier works by the authors.^22^, ^25^

The number of particle histories used in each DOSXYZnrc simulation, being between 1 and $2\;\times \;10^{9}$, was selected so that the statistical uncertainty in high‐dose voxels was approximately 1%. With MC model, the dose report mode was also $D_{m,m}$. In general, the simulation parameter selection was performed without compromising the calculation accuracy, which led to long calculation times (several thousands of CPU hours per plan). A 3D Savitzky‐Golay adaptive window digital filter was applied to denoise the MC dose distributions in the phantom study, to allow the point dose comparison (as suggested by Kawrakow^(26)^ and also applied by Teke et al.^(27)^), but not with the patient plan, to preserve the sharp changes in the intensity modulated VMAT dose distribution.

### C. Calculation grids based on phantom and patient images

The CT scanner, with imaging parameters FOV 55 cm, 120 kV, and 250 mA used in this study, was Toshiba Aquilion LB 16‐slice model (Toshiba Medical System, Otawara, Japan), which uses the 16‐bit depth for image pixels, yielding the extended CT scale. The CT‐based phantom and patient geometries were reconstructed with the CTCREATE code in DOSXYZnrc from sets of 1 mm thick and 3 mm thick CT slices, respectively, that were exported from the TPS. The CT number‐to‐material and density conversion curve, used both in the Eclipse TPS and in the MC calculations, were defined using the RMI Gammex 467 Tissue Characterization Phantom (Middleton, WI) with additional aluminum and titanium‐aluminum‐vanadium alloy (Ti6Al4V) inserts with known atomic compositions and densities. The transversal area of the phantom and the area of the high‐density material affect the acquired HU value for the high‐Z material (as in Coolens and Childs^(4)^), but the RMI phantom and the metallic rods used in the calibration were similar in cross‐sectional size, compared to the phantom and hip implant used in this study. Five different materials found in the default PEGS4 material library (AIR521ICRU, LUNG521ICRU, ICRUTISSUE521ICRU, ICRPBONE521ICRU, and AL521ICRU) and three additional materials (ICRP adipose tissue, ICRP cartilage tissue, and Ti6Al4V) created by the authors with PEGS4 utility found in the BEAMnrc code package, were assigned for the phantom, and patient voxels using the conversion curve and the corresponding cross section data for the materials were applied in MC dose calculation. In addition, for the PMMA material used in the phantom, PMMA521ICRU found in the default PEGS4 material library was used. All the above‐mentioned materials are implemented in the AXB algorithm material library and they were assigned in the calculations, as described in the following sections. The calculation grid sizes were 0.1 cm and 0.2 cm in the phantom study and with the patient plan, respectively, which were equivalent to the calculation grid sizes applied with the AXB algorithm and the AAA.

### D. The phantom study

In the first phase of this study, the AXB algorithm was compared against point dosimetry, film dosimetry, and full MC method in an anthropometric phantom with a unilateral hip implant made of Ti6Al4V alloy (1, 2). The intention of this phase was: 1) to assess the accuracy of the AXB algorithm in the presence of high‐Z material in CT‐based phantom; 2) to benchmark the MC model against measurements so that it can be used as reference in the second phase of this study; and 3) to quantify the effect of the CT artifacts in the calculation accuracy of the AXB algorithm and the MC model.

**Figure 1:**
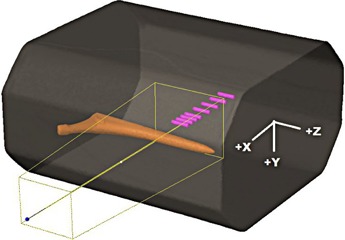
The phantom and a beam from the side, through the stem of the hip implant. The depths of the detectors are relative to the isocenter, the positive direction being towards the implant. The contoured cavities for the Farmer IC perpendicular to the beam are also presented.

**Figure 2:**
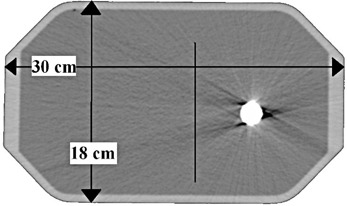
A phantom cross‐sectional CT slice at beam isocenter level with dimensions and the location of the measured dose profile are shown.

The phantom wall material was PMMA and it was filled with water. Transversal dimensions of the phantom were comparable to the pelvic region of an adult male (Fig. 2). A single 6 MV photon beam with field size 8 cm × 10 cm and the MLC retracted, was directed horizontally from the side of the phantom. The isocenter was located in the center of the phantom. The reason why the measurements were performed in the shadow of the stem, not the more clinically relevant cup of the implant, was that with this setup the CT artifacts were less pronounced and, thus, the actual computational differences were more easily identifiable. The measurement setup, the phantom with the implant, and Farmer IC detector cavity positions for depth dose measurements, are presented in Fig. 1. The IBA SFD was placed parallel to beam axis, the measurement points being at 0.7 mm from the tip of the detector. The vertical profile was measured at + 1.55 cm from the isocenter, which represents a clinically relevant location with respect to the prostate (Fig. 2).

The absolute point‐dose measurements were done according to the IAEA TRS 398 protocol.^(28)^ The doses were measured in the shadow of the stem of the implant with Scanditronix Wellhöfer FC65‐G Farmer‐type 0.6 cm^3^ IC (Farmer IC) and IBA SFD, which was calibrated against the IC using an intermediate field size $\left(5\;\times \;5\;{\text{cm}}^{2}\right)$. The measured dose values in the phantom represent the absorbed dose in water. Since with the AXB algorithm and the MC model the dose report mode is $D_{m,m}$, the measured values were converted to dose‐to‐medium by dividing the values by the average water‐to‐medium stopping power ratio. The phantom water was recognized by the algorithms as ICRUTISSUE521ICRU according to the HU value and, thus, the average water‐to‐medium stopping power ratio of 1.01 was selected, according to Siebers et al.^(29)^ In addition, a vertical dose profile was determined with the GAFCHROMIC (International Specialty Products, Wayne, NJ) EBT3 film. The film was attached to a plastic holder, tightening it parallel to beam axis and keeping it in water only for 2 min to minimize the immersion of water from the edges of the film. Two film sheets were irradiated in the same position and an average reading of both measurements was determined. The films were scanned with Epson Perfection V750 Pro flatbed scanner (Seiko Epson Corporation, Nagano, Japan) four days after the irradiation. During this time, the optical density stabilized and same time delay was used in film calibration. The calibration was performed at secondary standard dosimetry laboratory (SSDL) in Helsinki with ^60^Co radiation source. The scanning was done with 48‐bit color depth, using only the red channel for dose determination and blue channel for dust effect removal. First, the signal was corrected for the longitudinal and latitudinal distortion of the signal, caused by the scanner, by subtracting the determined transmission signal value of the unexposed film. The dose‐dependent latitudinal distortion of the signal, caused by polarization, was corrected by a function, which was determined with various flat dose levels. The dose values for the EBT3 measurements were normalized to the interpolated depth‐dose value from IBA SFD measurements by using the average of the EBT3 measurement values around the isocenter falling into the width of the IBA SFD active volume.

The calculations were performed with an original CT dataset and a corrected CT dataset, where constant HU values were assigned. With the original CT dataset, which with CT artifacts is shown in Fig. 2, the only assignment was that all the voxels with density larger than 3.0 g/cm^3^ were assigned as Ti6Al4V. It is a requirement by the AXB algorithm that such voxels have to have a material assigned and similar changes were made to actual HU values of the CT dataset to account for the assignment also in the MC calculations. This allows a fair comparison between the calculation methods. In the corrected CT dataset: 1) the implant was assigned to a constant HU value corresponding the density of the material (known density of $4.42\;g/{\text{cm}}^{3}$, corresponding to 7804 HU); 2) all the water was assigned to a constant HU value representing a mean HU value for the water in artifact‐free areas (0 HU); and 3) the walls of the phantom were assigned as PMMA material (120 HU). Corresponding calculated absolute dose values for the Farmer IC were determined by calculating mean values inside the contoured detector cavities on the both CT datasets. For the IBA SFD, since the thickness of the active volume is negligible (0.06 mm) in beam direction and two other dimensions are small as well, the calculated absolute dose values represented the values of one calculation grid voxel, which can be considered as point dose. Ultimately, measured values were compared to the calculated values, both in the original and in the corrected CT dataset, to assess the accuracy of the AXB algorithm, to benchmark the MC model, and to quantify the effect of the application of constant HU value assignment.

### E. The clinical demonstration with a patient plan

In the second phase of this study, the intention was to demonstrate whether the improved dose calculation accuracy of the AXB algorithm over the AAA can be transferred from the phantom to a clinical environment for prostate cancer patients or other patients with cancer in the pelvic region with a unilateral hip implant. Prior to dose planning, the constant HU value assignment was applied to the CT dataset that used extended CT scale: 1) the implant was contoured and assigned to Ti6Al4V based on the mean HU values (7804 HU, density $4.42\;g/{\text{cm}}^{3}$), and 2) the regions with over‐ and underestimated HU values were contoured with automatic tool and assigned HU value that was representative for the surrounding tissue (0 HU).

Since the Progressive Resolution Optimizer (PRO 3), which is applied for VMAT plan optimization, uses a simplified dose calculation model, it is not able to correctly account for high‐Z materials in the optimization process. Therefore, no VMAT beams should be passed through the implant and, thus, we applied static beams combined with arcs with avoidance sectors to the plan, which we denote as a hybrid VMAT technique plan. Two arcs (collimator angles 315° and 45°) were prepared, applying avoidance sectors for those beams‐eye‐view (BEV) projections, where the beam would pass through the implant. Three static 6 MV photon beam fields were set to cover the avoidance sector with regular gantry angle intervals. It was tested that three fields produced the best overall result, instead of smaller or larger number of fields of 6 MV or other energy (results not presented in this paper). In each field, MLCs were set to conform the planning target volume (PTV) shape, and combined dose contribution of the fields was first calculated. The dose distribution was normalized so that the maximum dose in the PTV was 0.5 Gy. The reason for this was that the avoidance sector in a VMAT‐only plan would cover approximately 25% of the whole arc, which equals to 0.5 Gy per fraction in common 39 × 2 Gy fractionation schedule. On the other hand, the authors noted that in several VMAT prostate plans with patients with no hip implant, the number of monitor units (MUs) from the typical avoidance sector part (for unilateral hip implant patients) is approximately 25% of the MUs of the whole arc. The dose distribution using static beams was then used as base dose plan for the two partial‐arc VMAT plan optimization, described above. Ultimately, the final hybrid VMAT plan consisted of two arcs with avoidance sectors and three static MLC‐shaped fields. Finally, the original plan was recalculated with the MC model and the AAA using the same number of monitor units (MUs).

The goal of the planning process was to achieve clinically acceptable dose distribution, with 78 Gy dose prescription to the PTV, so that at least 95% of the PTV volume would be covered with the 95% isodose and the maximum dose would be about 110% at maximum, preferably less. The dose in organs at risk (OAR) (rectum, posterior wall of the rectum, and bladder) was minimized, so that at least the following DVH criteria would be fulfilled: the volume of the rectum receiving 75 Gy/70 Gy/60 Gy/50 Gy was less than $5\%-10\%/20\%/35\%/60\%\;{(V}_{75}{/V}_{70}{/V}_{60}{/V}_{50})$, the maximum point dose to the posterior wall of the rectum was $50\;\text{Gy}\;\left(D_{50}\right)$, and the volume of the bladder receiving 65 Gy/50 Gy was less than $25\%/50\%(V_{65}/V_{50})$.

The CT datasets, structure sets, and dose distributions calculated with the AXB algorithm and the AAA were exported to CERR software, where also the MC‐recalculated dose distributions were imported. CERR, which uses MATLAB software (The MathWorks, Natick, MA) (version R2013a in this study), is a software package for the review and analysis of mainly radiotherapy planning data.^30^, ^31^ To quantify the accuracy of the AAA and the AXB algorithm, a DVH comparison was performed for the OARs with the criteria above, added with values at lower dose level for the bladder and the rectum and, in addition, near maximum ($D_{2}$) and minimum ($D_{98}$) doses that cover 2% or 98% of the PTV volume, respectively, and mean dose for $\text{PTV}\;({\text{PTV}}_{\text{mean}})$ were compared. Moreover, 3D gamma analysis tool within CERR software was applied between the MC model and the AXB algorithm to allow overall plan comparison, for which following threshold criteria were set: 2% (of maximum dose) in dose difference and 2 mm in distance‐to‐agreement (DTA) (2% / 2 mm). To minimize the effect of inherent noise present in MC‐based dose distributions on gamma analysis results, large number of particle histories were simulated in MC calculations to minimize the statistical uncertainty and regions with less than 15% of maximum dose were neglected in the gamma analysis calculation. The results were presented with the gamma agreement index (GAI), which is the ratio of the number of calculation points passing the gamma test and the number of all calculation points.

## III. RESULTS

### A. The phantom study

The dose‐to‐medium converted measured values by both the Farmer IC and the IBA SFD and calculated values for both the AXB algorithm and the MC model in both original CT and artifact‐corrected datasets are presented in 1, 2 and 3, 4, respectively. In Table 1 the results for the Farmer IC are presented, which reveals that on average, the AXB algorithm underestimates the measured dose, the deviations ranging from ‐1.1% to ‐2.2%. The largest deviations and the mean difference of ‐1.9% were for the calculations in the original CT dataset, whereas, for the corrected dataset, the mean difference is decreased to −1.5%. For the MC model, the overall discrepancies compared to measurements were smaller, the deviations ranging from −0.9% to +0.7%. The largest deviations, as with the AXB algorithm and the mean difference of −0.6%, were found with the original CT dataset, but, with the corrected dataset, the average overestimation decreased to only +0.5%. The dose underestimation characteristic for the AXB algorithm and the better congruence of the MC model with the Farmer IC measurements, especially in the corrected CT dataset, can also be seen in 3, 4.

**Figure 3:**
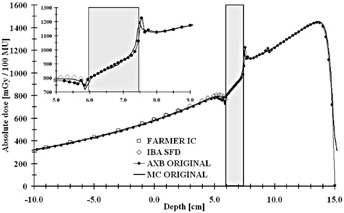
Measured (Farmer IC and IBA SFD) and calculated (the AXB algorithm and the MC model) depth‐dose values applying a 6 MV beam through the hip implant in the original CT dataset. The highlighted region represents the implant. The inset shows the magnification on the dose values in proximity of the implant and water interfaces.

**Figure 4:**
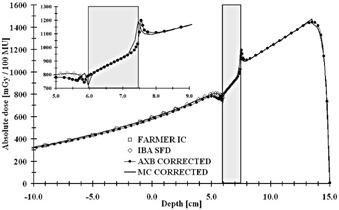
Measured (Farmer IC and IBA SFD) and calculated (the AXB algorithm and the MC model) depth‐dose values applying a 6 MV beam through the hip implant in the corrected CT dataset. The highlighted region represents the implant. The inset shows the magnification on the dose values in proximity of the implant and water interfaces.

In Table 2 are shown the results for IBA SFD. The AXB algorithm underestimates the measured and the MC‐calculated doses in the shadow of the implant, the mean differences being ‐2.4% and ‐2.5% for the original and corrected CT datasets, respectively, with the differences ranging from ‐5.5% to +2.2%. Again, for the MC model, the overall discrepancies compared to measurements were smaller, the deviations ranging from −3.1% to +0.1%. The largest deviations and the mean difference of −1.8% were found with the original CT dataset, but, with the corrected dataset, the average underestimation decreased to only −0.7%, even when including the measurement point closest to the implant, which is shown in the inset on Fig. 4, where the local difference is −3.1%. However, the AXB algorithm is congruent with the MC model upstream from the implant, as seen in 3, 4. In Fig. 3, the small disturbation in the calculated dose distributions upstream of the implant are due to the artifacts in the CT dataset. In Fig. 4, the small peak present at position 13.5 cm in the MC‐calculated depth dose curve represents the interface between the PMMA phantom wall and water. In both 3, 4 insets it can be seen that both the MC and the AXB algorithm model the beam propagation similarly inside the implant, but especially in the distal interface there are discrepancies of varying degree, also seen in 1, 2. In the proximal interface, the peak in the dose distribution caused by the increased backscatter from the high‐Z material to the lower density material is modeled similarly by both calculation methods.

**Table 1:** Measured and calculated absolute dose values (mGy/100 MU) in dose‐to‐medium for the Farmer IC. Difference is calculated by (calc.‐meas.)/meas.×100%. The depth corresponds to the distance from the isocenter level

*Depth (cm)*		*AXB Original*		*AXB Corrected*		*MC Original*		*MC Corrected*	
	*Measured*	*IC vol*.	*Diff*.	*IC vol*.	*Diff*.	*IC vol*.	*Diff*.	*IC vol*.	*Diff*.
‐9.00	344	338	‐1.7%	340	‐1.1%	342	‐0.5%	346	0.6%
‐7.00	388	380	‐2.2%	382	‐1.7%	385	‐0.9%	390	0.4%
‐5.00	436	428	‐1.9%	430	‐1.4%	433	‐0.7%	439	0.7%
‐3.00	492	482	‐2.0%	484	‐1.6%	488	‐0.8%	495	0.6%
‐1.00	553	544	‐1.7%	546	‐1.3%	551	‐0.4%	557	0.7%
0.00	590	578	‐2.1%	580	‐1.7%	585	‐0.9%	592	0.3%
1.00	627	615	‐2.0%	616	‐1.8%	623	‐0.7%	629	0.3%
2.00	664	654	‐1.5%	656	‐1.2%	663	‐0.2%	668	0.6%
Mean diff.			‐1.9%		‐1.5%		‐0.6%		0.5%

**Table 2:** Measured and calculated absolute dose values (mGy/100 MU) in dose‐to‐medium for the IBA SFD. Difference is calculated by (calc.‐meas.)/meas.×100%. The depth corresponds to the distance from the isocenter level

		*AXB Original*		*AXB Corrected*		*MC Original*		*MC Corrected*	
*Depth (cm)*	*Measured*	*SFD point*	*Diff*.	*SFD point*	*Diff*.	*SFD point*	*Diff*.	*SFD point*	*Diff*.
‐0.07	591	574	‐2.8%	574	‐2.8%	580.5	‐1.8%	587	‐0.6%
0.93	632	611	‐3.3%	611	‐3.3%	617.5	‐2.3%	623	‐1.4%
1.94	668	650	‐2.7%	650	‐2.8%	657.5	‐1.6%	662	‐0.9%
2.94	711	692	‐2.7%	691	‐2.8%	699.1	‐1.7%	704	‐1.1%
3.94	755	737	‐2.4%	737	‐2.4%	745.6	‐1.3%	752	‐0.4%
4.44	779	759	‐2.6%	760	‐2.5%	769.6	‐1.2%	773	‐0.8%
4.94	800	777	‐2.9%	779	‐2.6%	788.7	‐1.4%	797	‐0.4%
5.14	805	781	‐3.0%	781	‐3.0%	793.1	‐1.5%	803	‐0.3%
5.34	809	776	‐4.0%	776	‐4.1%	792.4	‐2.0%	807	‐0.2%
5.54	806	766	‐4.9%	762	‐5.5%	788.7	‐2.1%	807	0.1%
5.74	795	782	‐1.7%	782	‐1.7%	774.6	‐2.6%	794	‐0.2%
5.94	769	786	2.2%	783	1.8%	752.3	‐2.2%	745	‐3.1%
Mean diff.			‐2.4%		‐2.5%		‐1.8%		‐0.7%

**Figure 5:**
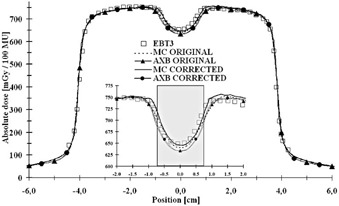
Measured (EBT3) and calculated (the AXB algorithm and the MC model) profiles applying a 6 MV beam through the hip implant in the original and corrected CT datasets at a distance 1.55 cm from the isocenter. The inset shows the magnification on the dose values in the shadow of the implant.

In Fig. 5 it can be observed that the EBT3 measurement values are close to the dose values of the MC model in the corrected CT dataset, even in the shadow of the implant, where the AXB algorithm underestimates the dose in both the original and the corrected CT datasets. The dose gradient in the corrected CT dataset for the MC model is larger than in the original dataset. With the AXB algorithm, the dose distributions in the shadow of the implant are practically identical in both CT datasets. The only differences with the AXB algorithm occur in the high‐dose region where, due to artifacts in the original CT dataset, the dose distribution is slightly perturbed, which is also the same with the MC model.

### B. The clinical demonstration with a patient plan

**Figure 6:**
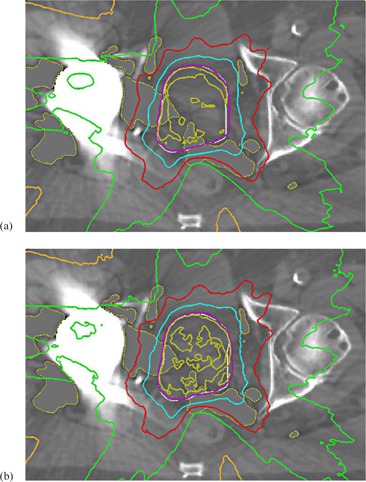
The isodose curves for clinical patient plan demonstration calculated both by the AXB algorithm (a) and the MC model (b). Isodose levels from outermost curve are 10%, 30%, 50%, 70%, 95%, and 100% of prescribed dose (PTV white). The constant grey areas represent where the HU correction is applied.

**Figure 7:**
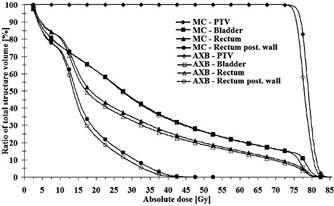
The DVH curves for the patient plan calculated both by the AXB algorithm and the MC model.

The results for the clinical patient plan demonstration are presented in Figs. 6(a)–6(b), Table 3, and Fig. 7. In Figs. 6(a) and (b) are shown the isodoses for hybrid VMAT technique plans, calculated both by the AXB algorithm and the MC model. For the isodoses, the congruence between the AXB algorithm and the MC model is very good, both in high‐dose level around and inside the PTV, but especially in the areas of lower dose levels. Relatively good congruence is achieved also between the AAA and the MC model in the PTV and OARs, but discrepancies are seen in the vicinity of and inside the implant. To quantify the accuracy of the algorithms in the tissue/high‐Z material interface, maximum doses in a layer ranging between 3 mm outside and 1 mm inside the interface were determined. The maximum doses were 38.4 Gy, 38.6 Gy, and 36.9 Gy for the MC model, the AXB algorithm, and the AAA, respectively, which means the discrepancies are 0.5% and 3.9% for the AXB algorithm and the AAA, respectively, when compared to the MC model. The DVH curves for the dose distributions presented in Fig. 7 support the results shown by the isodose curves in Figs. 6(a) and (b). In the DVH data for the PTV, rectum and the rectum posterior wall, the AXB algorithm produces slightly smaller dose values than the MC model, whereas for the bladder the DVH curves are similar, except at the highest dose levels. In Table 3, the results are shown quantitatively. The DVH values for the AAA (see Table 3) are omitted in Fig. 7 due to small discrepancies to the results of the AXB algorithm. The GAI value, 99.01%, verifies that the consistency between the MC model and the AXB algorithm is not compromised by the presence of the high‐Z material.

**Table 3:** Calculated DVH values for plans using the AAA, the AXB algorithm, and the MC model

		*AAA*	*AXB*	*MC*
PTV	$D_{2}$	104.7%	104.8%	105.9%
	$D_{98}$	95.2%	95.6%	97.0%
	$D_{\text{mean}}$	78.0 Gy	78.0 Gy	79.1 Gy
Bladder	$V_{65}$	18.2%	18.2%	18.4%
	$V_{50}$	26.4%	26.1%	26.4%
	$V_{40}$	34.1%	33.7%	34.1%
Rectum	$V_{75}$	6.2%	6.4%	7.4%
	$V_{70}$	9.5%	9.5%	10.5%
	$V_{60}$	14.1%	14.1%	15.1%
	$V_{50}$	18.8%	18.6%	19.8%
	$V_{20}$	45.8%	44.5%	47.0%
Rectum post. wall	$D_{\text{max}}$	47.2 Gy	46.5 Gy	48.2 Gy

## IV. DISCUSSION

### A. The phantom study

The results for the phantom study indicate that the full MC simulations are the most accurate method to calculate dose distributions in phantom volumes containing high‐Z materials, as has been shown in several earlier studies. The results of the MC model used in this study are very close to measured values, deviations mostly being approximately ±0.5% (1, 2). The AXB algorithm was capable of producing dose distributions close to the MC model and measurements. This result is consistent with the study by Lloyd and Ansbacher,^(11)^ in which the AXB algorithm showed only small deviations in the upstream and downstream interfaces of high‐Z material, when compared to GAFCHROMIC EBT2 point measurements and full MC model. In the lateral dose profile through the high‐Z insert, the AXB algorithm has also been congruent with the MC model, which is consistent with the results of our study. The current results are also consistent with findings by Failla et al.^(10)^ The small dose underestimation observed for the AXB algorithm in this study, when comparing to the measurements and the MC model, is most probably due to the slightly different alignments of the dose calculation grids and CT datasets between the two calculation methods. This might have led to small increase in the thickness of the high‐Z material and, thus, to increased attenuation and lower dose values in the shadow of the implant. In the light of our results, the validity of the MC model used in this study as a reference method is confirmed for the dose calculations in the presence of high‐Z material.

The effect of the constant HU value assignment, combined with the extended CT scale, was noticeable in the MC model calculation results, even in the simple phantom geometry used in our study. The calculated dose values in the corrected CT dataset were closer to the measured values. The reason for this was the partial volume effect and HU value blurring in the high‐Z material interface, which artificially increased the dimensions of the implant and resulted in increased dose attenuation in the original CT dataset. Regardless, the extended CT scale allowed the accurate high‐Z structure delineation and material assignment. Therefore, as done in the clinical patient plan demonstration, the utilization of the constant HU value assignment with the extended CT scale is recommended when the beams traverse the implant, as shown by Glide‐Hurst et al.^(5)^ and Bazalova et al.,^(32)^ for example.

An important detail in the calculated dose distributions is the peak in the proximal interface, caused by the increased backscatter from the high‐Z material to the lower density material. As shown by Failla et al.^(10)^ and Lloyd and Ansbacher,^(11)^ the AXB algorithm is the first commercial algorithm able to model this interface phenomenon. In this study it is also shown that the AXB algorithm and the MC model produce similar peaks in the dose distribution and, therefore, the AXB algorithm can be used for dose calculations where beams traverse high‐Z material, as long as the material is identified from HU values, correct material is assigned, and some level of MAR is applied.

#### B. The clinical demonstration with a patient plan

The results of the phantom study attest to the accuracy of the AXB algorithm when beams traverse high‐Z material, which allows the treatment plan to include such fields without introducing large uncertainties in the resulting dose distribution. For example, in case of treatment plans for patients with cancer in the pelvic region, this enables the use of beam angles that account for ∼25% of all the coplanar beam directions in plans with unilateral hip implants and ∼50% in plans with bilateral hip implants.

In this study, a clinical patient plan demonstration was performed. The accuracy of the AXB algorithm was evaluated using a hybrid VMAT technique for the treatment planning of a prostate cancer patient with a unilateral hip implant. The original plan was calculated with the AXB algorithm and recalculated with the MC model and the AAA. Comparison of the isodose curves in the implant and elsewhere confirms that the deviations between the MC model and the AXB algorithm were small, the MC model producing slightly higher doses. The good congruence is confirmed with the gamma analysis results. For the AAA, the only notable discrepancies were in the vicinity of and inside the implant, which may only partly be justified by the dose‐to‐medium vs. dose‐to‐water difference. This means that the AXB algorithm is a reliable dose calculation algorithm also for patient plans with hip implants that contain beams traversing the implant, but the use of AAA is not encouraged. For such plans, in addition to the inability of commercial dose calculation algorithms (especially type ‘a’) in the past to produce accurate dose distributions in the target volume and in OARs in central pelvis, another concern has also been the peak‐shaped dose in the interface between the high‐Z material and bone. However, the discrepancies at the interface with the AAA are smaller than expected from the phantom studies applying a single beam.^(11)^ The reason for this is that the dose peak underestimation for the beam directions, for which the interface acts as an upstream surface, is compensated by the dose overestimation for the beam directions, for which the interface acts as a downstream surface. This phenomenon is obvious especially for the plan in this study, where beams enter the patient quite uniformly from all directions in the transversal plane.

Excessive doses in the interface region would lead to radiation complications, such as bone necrosis and problems in the implant fixation. In the example patient plan in this study, the maximum dose to the tissue/high‐Z material interface was not more than 38.6 Gy (AXB), mostly clearly below this. This means that if 50 Gy is considered a risk limit for radiation complications^(3)^ and the AXB algorithm correctly calculates the dose distribution in the interface, there are no constraints to apply the presented hybrid technique or complex treatment plans produced with some other modern treatment technique for radiotherapy of the pelvic region for patients with hip implants. If dose escalation in prostate radiotherapy continues, the tissue/high‐Z material interface dose levels have to be carefully monitored in order to keep them under the risk limit. If beams are to be applied through the implant, each different implant material should be tested separately, since the dose calculation accuracy depends strongly on the CT number‐to‐material and density conversion curve and material assignment. In addition, the dose calculation grid size has to be as small as possible in order to correctly calculate the sharp dose gradients in the tissue/high‐Z material interface. As the results show, beams traversing the implant are no longer prohibited due to limited dose calculation accuracy, and, therefore, it is intuitive to hypothesize that such beams would be favorable especially in a patient plan with bilateral implants, since, in that case, about 50% of beam directions should be avoided when using other techniques. The clinical benefit, combined with the utilization of MAR techniques, should be quantified with further patient plan studies.

## V. CONCLUSIONS

In this study, the full MC model was shown to produce dose distributions on average within 0.7% against the measurements in water phantom with beams traversing high‐Z material. The dose distributions calculated by the AXB algorithm showed small deviations, on average within 2.5%, when compared to measurements and the MC model, but the performance was considered acceptable. The full MC model and the AAA were applied to recalculate a clinical example patient plan with a unilateral hip implant using a hybrid VMAT technique introduced in this study. This was the first time to report on the accuracy of the AXB algorithm compared with the full MC model with plans using VMAT technique. The agreement between the AXB algorithm and the full MC model was very good, which verifies the accuracy of the AXB algorithm for patient plans with beams traversing high‐Z material. Therefore, the AXB algorithm can be used for clinical purposes in such cases with confidence. However, the use of the AAA is not encouraged, due to increased uncertainty in dose distributions in the vicinity of and downstream from the implant due to inaccurate modeling of the backscatter from and beam attenuation in the implant, respectively.

## ACKNOWLEDGMENTS

The authors would like to thank T. Popescu and J. Lobo from British Columbia Cancer Agency, Vancouver Centre, for their assistance in extracting plan parameters, which were needed in MC simulations, from the Eclipse TPS. The authors declare that they have no conflicts of interest. The authors alone are responsible for the content and writing of the paper.
